# Declarations: management of a pulmonary arteriovenous fistulae by uniportal video‐assisted thoracoscopic surgery: a case report

**DOI:** 10.1186/s12893-021-01103-8

**Published:** 2021-02-23

**Authors:** R. Li, Y. Zhou, S. Kang, F. Kong, L. Guan, Y. Zhao, X. Yin

**Affiliations:** 1 Department of Thoracic Surgery, The First Affiliated Hospital of Kunming Medical University, Kunming, 650032 Yunnan China; 2 Department of Imaging, The First Affiliated Hospital of Kunming Medical University, Kunming, 650032 Yunnan China

**Keywords:** Pulmonary arteriovenous fistula, 3D-CT reconstruction, Uniportal VATS, Case report

## Abstract

**Background:**

A pulmonary arteriovenous fistula (PAVF) is a rare condition that is associated with pulmonary arteriovenous malformation (PAVM). Few reports have described managing PAVMs using uniportal video-assisted thoracoscopic surgery (VATS).

**Case presentation:**

A 13-year-old child with PAVF in the left inferior pulmonary artery was treated by uniportal VATS with left lower lobectomy. After surgery, hemoptysis did not recur and there were no postoperative complications. Six months after the operation, postoperative review of computerized tomography showed no recrudescence of PAVF.

**Conclusions:**

PAVF is a rare case that should be diagnosed and treated early. 3D- computerized tomography (CT) reconstruction is useful for diagnosis and preoperative assessment. The case shows that PAVF can be managed with uniportal VATS.

## Background

A pulmonary arteriovenous fistula (PAVF) is a rare condition, first described by Churton in 1897 [1], that is associated with pulmonary arteriovenous malformation (PAVM), which allows abnormal direct communication between the pulmonary arteries and pulmonary veins [2]. The most common cause of PAVM is hereditary hemorrhagic telangiectasia, also known as Osler–Weber–Rendu syndrome [3]. Patients without obvious symptoms account for approximately 57 % of PAVF cases in the early stage of the disease. However, PAVF can also exhibit diverse symptoms, including repeated hemoptysis, nosebleeds, difficulty catching breath, an increase in hemoglobin levels, and hemoptysis, which can lead to sudden fatal rupture of the veins [4, 5]. Patients with these symptoms are treated with interventional therapy [6] or surgical procedures including lung wedge resection, lobectomy, and pneumonectomy [7, 8]. The following is a case report of a 13-year-old patient with PAVF in the left inferior pulmonary. The patient, who exhibited hemoptysis and dyspnea, was diagnosed by preoperative 3D-computed tomography (3D-CT) reconstruction and treated by uniportal video-assisted thoracoscopic surgery (VATS).

## Case presentation

**Fig. 1:**
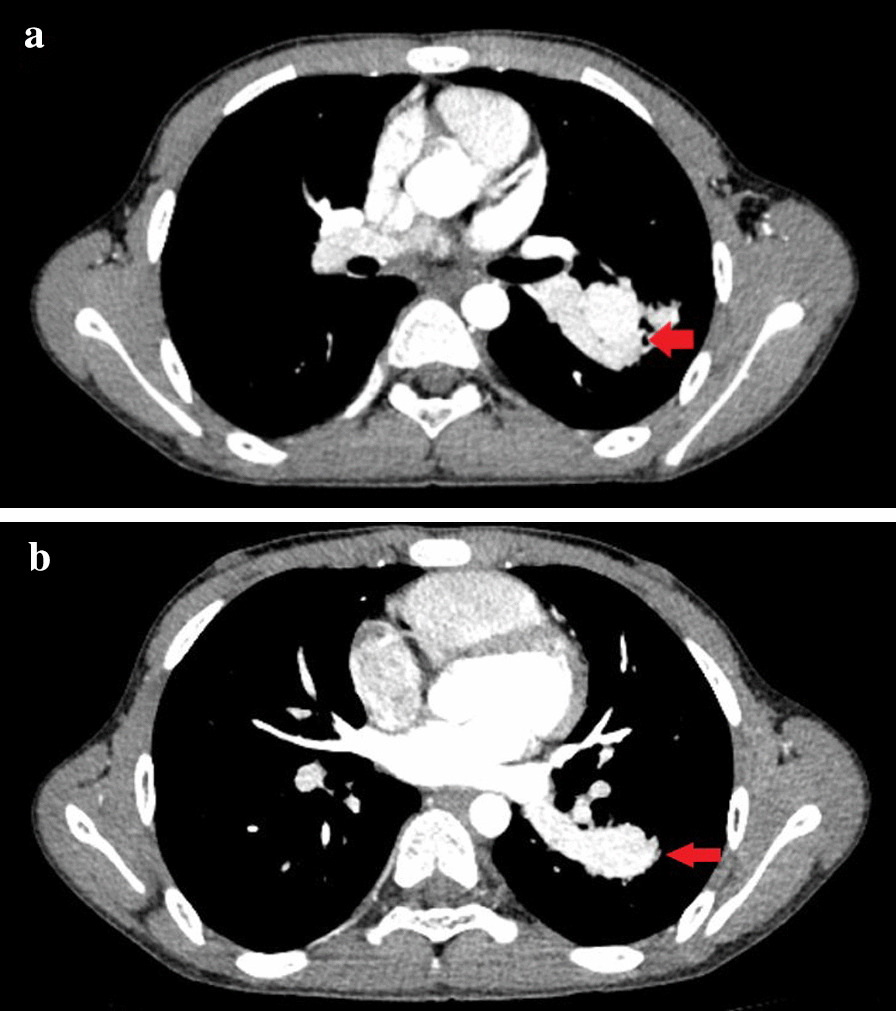
**a**, **b** CTA scan showed a large 3.8 × 3.2 cm lesion in the left lower lobe. (Mediastinal window)

**Fig. 2:**
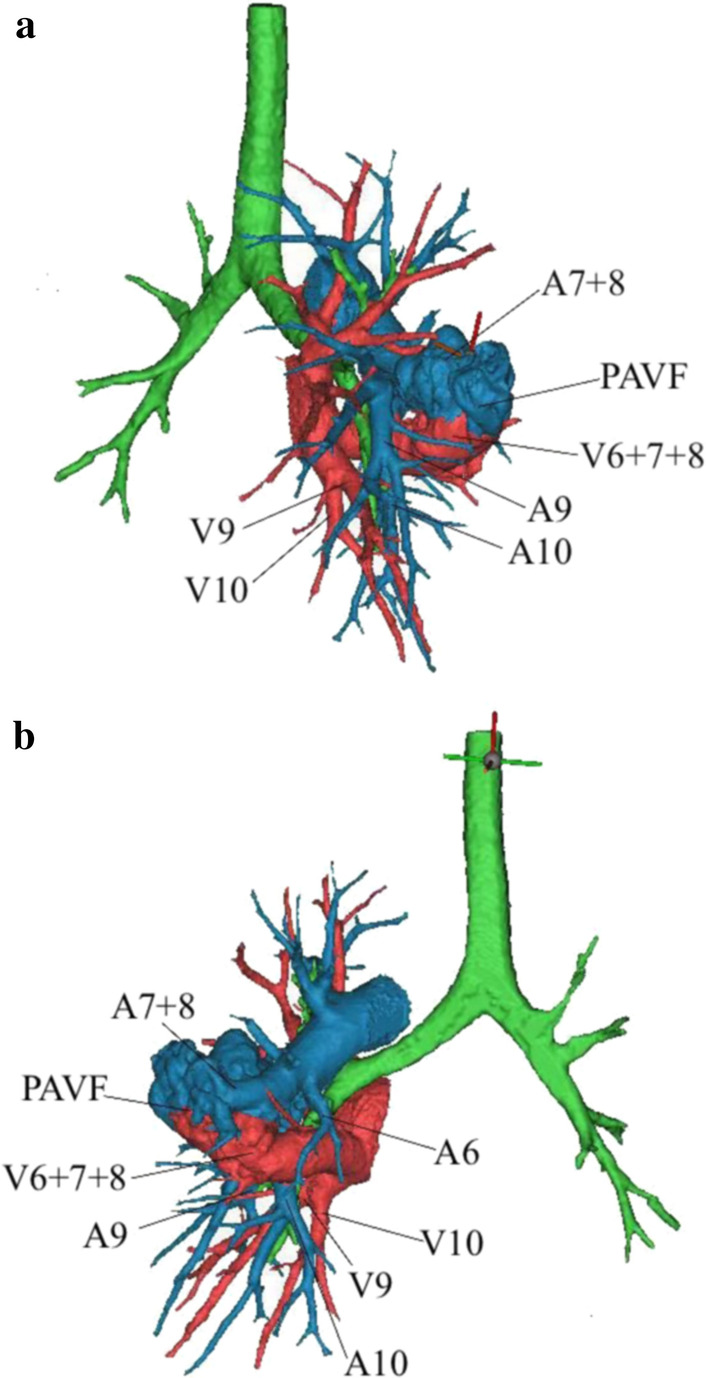
**a**, **b** 3D-CT reconstruction demonstrated that the lesion was an abnormal direct communication between left inferior pulmonary arteries and veins. (**a** Anterior view; **b** Posterior view)

A 13-year-old child developed symptoms of mild hemoptysis and dyspnea that persisted for more than two months and did not respond to oral medication. Physical examination showed only a mild cyanosis of lips, with no other obvious findings. The patient had no relevant previous medical history and there was no relevant family history. Chest radiography indicated that a mass was located in the left inferior pulmonary. Non-enhanced chest computerized tomography (CT) and subsequent computerized tomographic angiography (CTA) confirmed a large 3.8 × 3.2 cm lesion in the left lower lung lobe (Fig. 1a, b). A 3D-CT reconstruction [9] made by Mimics Medical 21.0 confirmed what appeared to be a PAVM or PAVF before surgery, which was directly connected between A^7–8^ of left lower pulmonary arteries and V^6–8^ of left lower pulmonary veins not feeder vessels (Fig. 2a, b). Bronchoscopy indicated that normal tracheobronchial anatomy was affected by the endobronchial lesion and by airway compression. A routine blood examination showed that hemoglobin was 113.0 g (normal range: 120–172), hematocrit 47 % (normal range: 36–50 %), arterial blood gas PaO_2_ 59 mmHg (normal range: 83–108) and SO_2_ 93 % (normal range: 95–98 %).

**Fig. 3:**
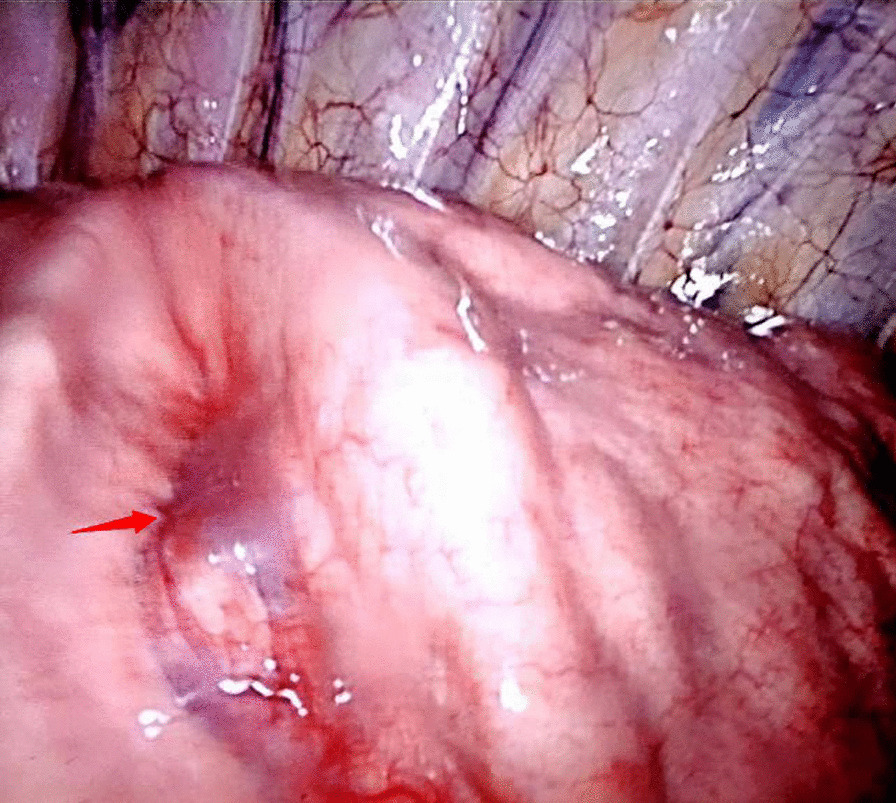
A dark blue mass was found on the left interlober fissure

**Fig. 4:**
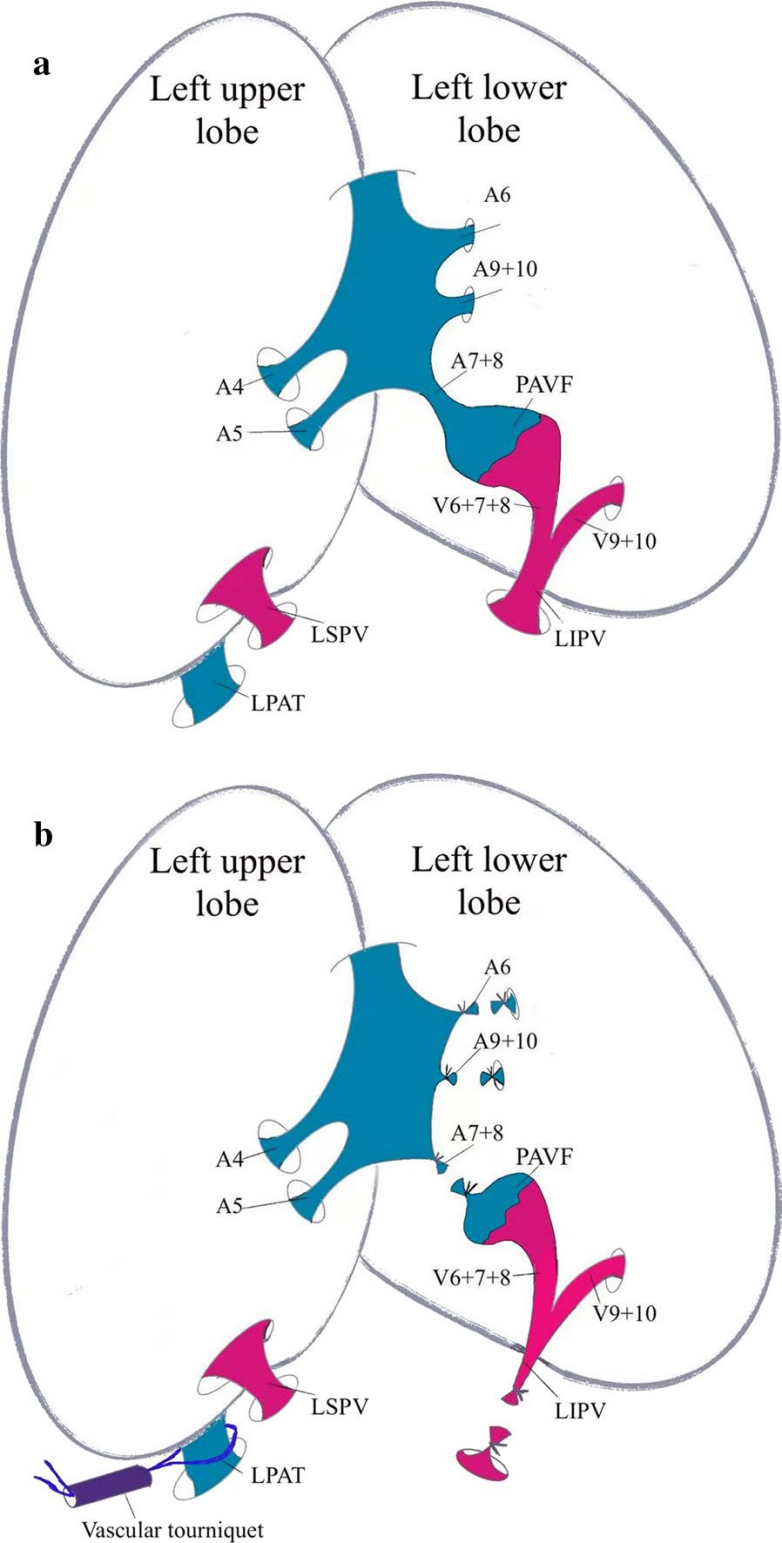
Sketch profile of the pulmonary arteriovenous fistula

**Fig. 5:**
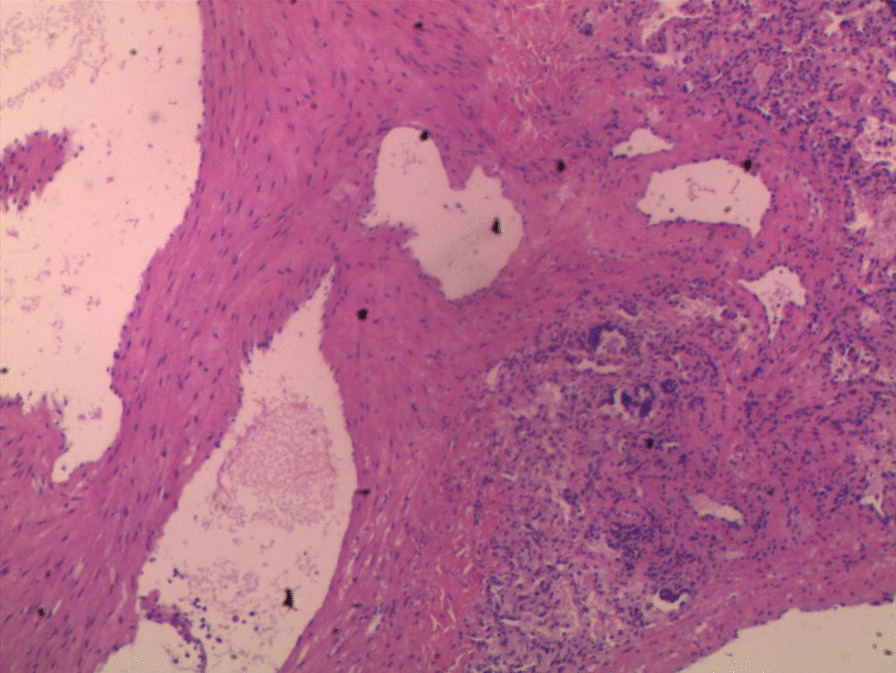
A pathological diagnosis of PAVF

Uniportal VATS was carried out through a 3 cm incision in the fifth intercostal anterior-axillary space. A dark blue mass on the left interlobar fissure was found via thoracoscopy and was concluded to be a PAVM (Fig. 3). The lobectomy began by releasing pleuropulmonary adhesions and the inferior pulmonary ligament and dissecting the aortopulmonary to expose the left pulmonary arterial trunk (LPAT), which was controlled proximally by using a vascular tourniquet. The major fissure was then divided to identify the left inferior pulmonary artery. The left inferior pulmonary vein (LIPV) and the left superior pulmonary vein (LSPV) were exposed after dissecting the hilum of left pulmonary arteries. The large PAVM was directly connected between A^7–8^ of left lower pulmonary arteries and V^6–8^ of left lower pulmonary veins, and this was consistent with the results of preoperative 3D-CT reconstruction. The left inferior pulmonary artery and vein were ligated using staplers (Fig. 4a, b). The bronchus of the left lower lobe was then clamped by using staples and removed during the operation. A pathological diagnosis of PAVF was confirmed (Fig. 5).

**Fig. 6:**
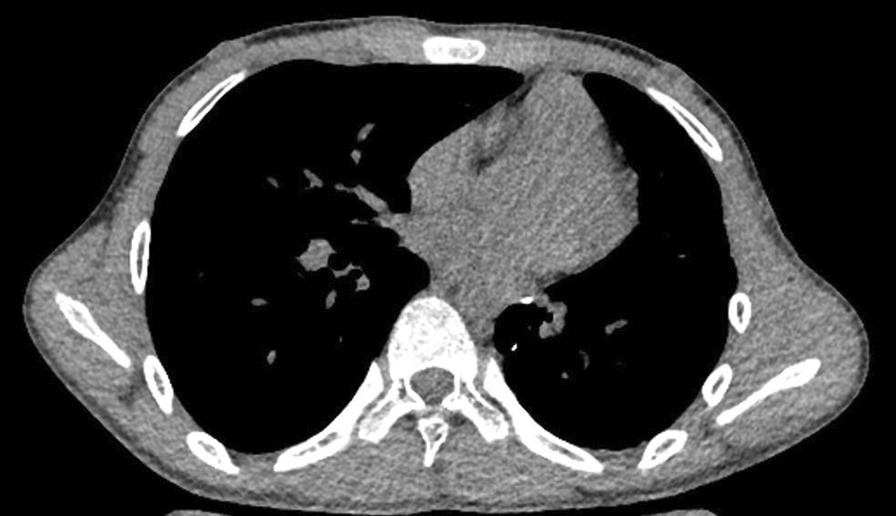
Postoperative review of computed tomography showed no recrudescence of PAVF. (Mediastinal window)

Hemoptysis and postoperative complications did not occur after surgery. The tube was removed after reviewing the chest X-ray film for pneumothorax and abnormal pleural effusion. The patient was discharged from hospital five days after surgical resection. Six months after the operation, postoperative review of computed tomography showed no recrudescence of PAVF (Fig. 6).

## Discussion and conclusions

The condition of PAVF occurs mainly due to defects in pulmonary capillary development. Abnormal communications between pulmonary arteries and veins form a right-to-left shunt causing blood flow without oxygenation and an increase in red cells. Complications may be caused by PAVMs due to direct intercommunication between pulmonary arteries and pulmonary veins, including exertional dyspnea, swirl, cyanopathy, polycythemia, bacterial infections, and brain abscesses. It can appear as a mass on routine chest radiography and abnormal direct communication between pulmonary arteries and pulmonary veins on CT or 3D-CT.

Appropriate therapies for PAVF are interventional therapy or surgical treatment. As in this case, a patient with serious PAVM and hemoptysis should be treated to prevent possible severe dyspnea due to life-threatening hemorrhage and hypoxemia caused by PAVM. In this case, because the diameter of the PAVF was limited to a lobe and was larger than 2 cm, with a.

feeding artery over 3 mm in diameter, it was decided that interventional therapy would not solve the problem and that a surgical procedure should be chosen if the operative risk could be controlled. Because the PAVF of the patient was located deeply, it was hard to remove using transcatheter embolotherapy and lung wedge resection. Therefore, left lower lobectomy was selected.

Three points were analyzed to reduce the thoracoscopic surgical risk during this operation. The 3D-CT reconstruction was used to confirm the structure of the PAVM before the operation. Preoperative simulation based on 3D-CT reconstruction is useful for accurate diagnosis, and in this case, 3D-CT reconstruction revealed a huge malformed hemangioma that was directly connected between the trunk of left lower pulmonary arteries and the left lower pulmonary veins not feeder vessels, which was coincident with the intraoperative situation. The use of 3D-CT reconstruction also showed the anatomical variation of the blood vessels, so life-threatening hemorrhage could be avoided during surgery. Risk analysis through 3D-CT reconstruction indicated that the left pulmonary trunk should be controlled proximally by using a vascular tourniquet to avoid uncontrolled arterial bleeding. This control of the left pulmonary trunk allowed the distally involved pulmonary parenchyma to be safely resected. More importantly, precise patient positioning for thoracoscopic pulmonary lobectomy allowed a suitable surgical plan for the operating surgeon and reduced the risks involved in thoracoscopic surgery. Few cases of PAVF have been published, so no outcome data were available for this surgical procedure. Uniportal VATS was performed for the PAVF in this case, because it was a secure and feasible technique in thoracic surgery and provided a better surgical field. To reduce surgical trauma, there was only one surgical hole. As such, there were fewer complications, less pain, and, consequently, a shorter postoperative hospital stay [10].

In general, PAVF is a rare case which should be diagnosed and treated early. 3D-CT reconstruction may be useful for diagnosis and preoperative assessment. The case could provide the experience and feasibility for PAVF managed with uniportal video-assisted thoracoscopy.

## Data Availability

The data and materials sets support the conclusions of this article.

## Abbreviations

PAVF: Pulmonary arteriovenous fistulae
PAVM: Pulmonary arteriovenous malformation
3D-CT: Three dimensional-computed tomography
VATS: Video-assisted thoracoscopic surgery
CT: Computed tomography
CTA: Computed tomograhy angiograhy
LPAT: Left pulmonary arterial trunk
LIPV: Left inferior pulmonary vein
LSPV: Left superior pulmonary vein
